# Differential processing of nociceptive input within upper limb muscles

**DOI:** 10.1371/journal.pone.0196129

**Published:** 2018-04-25

**Authors:** Nathanial R. Eckert, Brach Poston, Zachary A. Riley

**Affiliations:** 1 Department of Kinesiology, University of Indianapolis, Indianapolis, IN, United States of America; 2 Department of Kinesiology and Nutrition Sciences, University of Nevada-Las Vegas, Las Vegas, NV, United States of America; 3 Department of Kinesiology, Indiana University-Purdue University, Indianapolis, IN, United States of America; The Ohio State University, UNITED STATES

## Abstract

The cutaneous silent period is an inhibitory evoked response that demonstrates a wide variety of responses in muscles of the human upper limb. Classically, the cutaneous silent period results in a characteristic muscle pattern of extensor inhibition and flexor facilitation within the upper limb, in the presence of nociceptive input. The aims of the current study were: 1) to primarily investigate the presence and characteristics of the cutaneous silent period response across multiple extensor and flexor muscles of the upper limb, and 2) to secondarily investigate the influence of stimulation site on this nociceptive reflex response. It was hypothesized that the cutaneous silent period would be present in all muscles, regardless of role (flexion/extension) or the stimulation site. Twenty-two healthy, university-age adults (14 males; 8 females; 23 ± 5 yrs) participated in the study. Testing consisted of three different stimulation sites (Digit II, V, and II+III nociceptive stimulation) during a low intensity, sustained muscle contraction, in which, 7 upper limb muscles were monitored via surface EMG recording electrodes. Distal muscles of the upper limb presented with the earliest reflex onset times, longest reflex duration, and lowest level of EMG suppression when compared to the more proximal muscles, regardless of extensor/flexor role. Additionally, the greatest overall inhibitory influence was expressed within the distal muscles. In conclusion, the present study provides a new level of refinement within the current understanding of the spinal organization associated with nociceptive input processing and the associated motor control of the upper limb. Subsequently, these results have further implications on the impact of nociception on supraspinal processing.

## Introduction

The cutaneous silent period (CSP) serves as nociception triggered inhibition that provides momentary inhibition of voluntary muscle activity, in response to nociceptive activity transduced via Aδ and/or nociceptive C-afferent fibers to the spinal motorneuron pool through direct or indirect synaptic connections [1–4]. This action presents a reciprocal action to the nociceptive flexor withdrawal response (FWR) which serves as an ‘excitatory reflex’ increasing activity in primary flexor muscles [5]. When taken into context, the interplay between the CSP and FWR can clearly be seen. For example, if nociceptive input is generated while reaching forward with the upper limb, the CSP will momentarily halt the forward action through inhibition, while the FWR will activate the primary flexor muscles to remove the limb from potential harm via facilitation [5–7]. Therefore, the CSP can be thought of as a protective spinal reflex suppressing voluntary activity in muscles in an effort to reduce further activity resulting in an increase of nociceptive activity [4].

When evoked by strong electrical stimulation of the digits, the inhibitory evoked CSP response demonstrates a wide variety of responses in muscles of the human upper limb. For example, stimulation of digit II provides a powerful silent period within the abductor pollicis brevis (APB) muscle, nearly eliminating muscle activity for upwards of ~80ms [4]. Similar to the effect observed in the hand, some proximal muscles of the stimulated limb also show periods of inhibition, albeit shorter in duration. However, certain muscles such as the biceps brachii and deltoid muscles have been reported to show facilitation in response to stimulation, via the FWR response [2, 3, 6, 8, 9]. Accordingly, two main conclusions can be taken from these results: 1) the distal muscles of the hand show the most pronounced and longest duration of the CSP; and 2) the recorded responses to noxious input in the proximal muscles of the upper limb can be mixed. These two conclusions are consistent with studies that have shown that the CSP is modulated in reaching and grasping to assist grasp release and initiate a halting action on forward reaching movements [10, 11].

Early explanations of the CSP discussed a characteristic pattern of inhibition within extensor muscles with this response followed by facilitation of flexor muscles of the upper limb, presumably through the FWR [2, 7, 12]. It has been speculated that this pattern of inhibition and facilitation may serve to limit reaching away from the body and initiate the retraction of the upper limb towards the body. Ideally, this pulls the limb away from a potential noxious stimulus in the environment (e.g. touching a hot stove). This particular pattern of inhibition and facilitation, however, may be more complex than previously thought, as inhibition can be demonstrated within both flexor and extensor muscles, within various studies [3, 4, 7, 10, 11, 13]. From a movement perspective, the ability to produce inhibition within both extensor and flexor muscles becomes critical as, inhibition of both the flexor and extensor muscles could be required to remove the upper limb from potentially harmful environments, as seen in reaching and grasping. Therefore, it seems that the complexity of the CSP may include different patterns of extensor and flexor inhibition within, and across, the upper limb.

The current study sought to improve upon the current understanding of the CSP response in the upper limb by addressing two aims: 1) primarily investigate the presence and characteristics of the CSP response across multiple extensor muscles and flexor muscles of the upper limb, and 2) secondarily investigate the influence of multiple stimulation sites on the reflex response. The examination of the CSP response across the muscles of the upper limb will provide evidence to support or potentially refute previously accepted beliefs on the CSP while providing a more in-depth analysis on the organization of the CSP response across the upper limb in humans. It was hypothesized that the CSP would be present in all muscles, regardless of the role of the muscle (flexion/extension) or the stimulation site, which would demonstrate a different level of spinal organization than previously described.

## Materials and methods

### Participants

Twenty-two healthy, university-age adults (14 males/ 8 females; 23 ± 5 yrs) participated in the study. Subjects were self-reportedly free from any neurological disorders or other upper limb musculoskeletal impairments. Each subject provided written informed consent prior to participating. The protocol was approved by the Indiana University Institutional Review Board and was performed in accordance with the Declaration of Helsinki.

### Experimental protocol

#### Apparatus and testing environment

**Fig 1:**
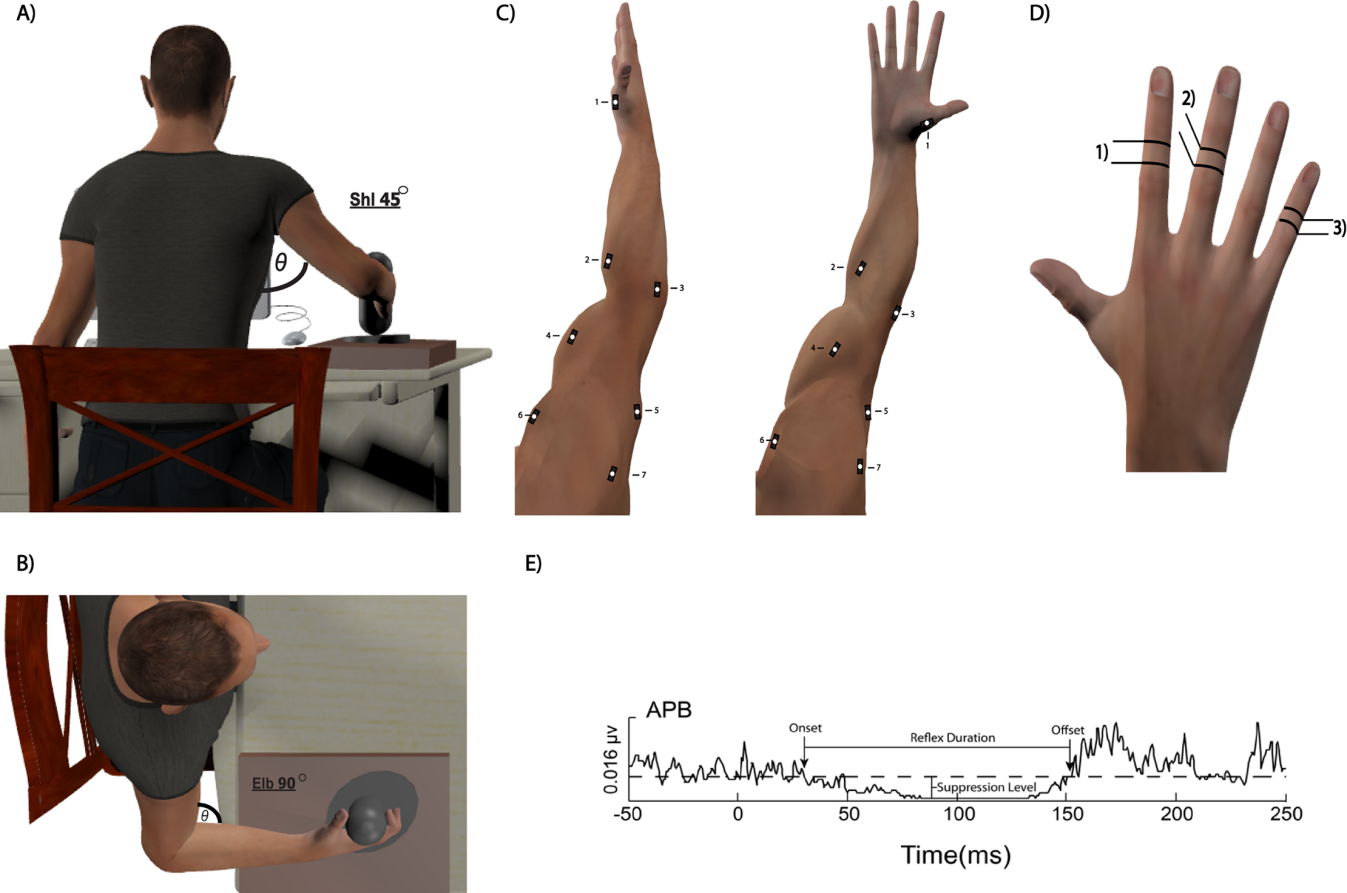
Representation of the data collection process using the custom made arm setup (A-B) along with EMG placement (C) and a representative trace of the identified specific reflex response variables (E).

Electrical stimuli were delivered using a computer-controlled Digitimer DS7AH constant current electrical stimulator (Digitimer LTD, England, UK). Electromyography (EMG), or muscle activity, was recorded on a 16-channel Bagnoli EMG system (Delsys Inc, MA, USA) using a bipolar montage of rectangular single differential bar electrodes (1cm x 1cm x 1mm). EMG activity was recorded from the abductor pollicis brevis (APB), flexor carpi radialis (FCR), extensor carpi radialis (ECR), biceps brachii long head (BIC), triceps brachii lateral head (TRI), anterior deltoid (AD), and posterior deltoid (PD) muscles of the right arm (Fig 1C:1–7). Electrodes were placed in accordance with previously established site standards and prepped with the skin overlying each muscle cleaned prior to affixing the electrode over the individual muscle belly, parallel with the orientation of the respective muscle’s fibers [14]. A single common ground electrode was placed over the spinous process of C7. Each subjects right arm was positioned, unrestricted, in a custom arm apparatus with the elbow flexed at 90°, while maintaining ~45° abduction of the shoulder from the torso, and the hand grasping a handle, fully grasped, thumb wrapped around, or through “cupping the hand” depending on the muscle action necessary, attached to a tri-axial force transducer (JR3 Inc., CA, USA) (Fig 1A and 1B). All testing was conducted with the subject seated upright, the upper extremity supported but not restricted, and with visual feedback provided on a computer monitor in front the subject. All data was processed and stored using Spike2 software (CED, Cambridge, UK).

#### Study procedures

Subjects were first asked to complete three maximal isometric voluntary contractions (MVCs) separated by 3 minutes, for each recorded muscle, by isometrically contracting that muscle in its direction of force (i.e. flexion or extension) against an immovable vertical handle grasped by the hand, fully grasped, thumb wrapped around, or through “cupping the hand” depending on the muscle action necessary. The MVC with the highest unrectified peak EMG value was then used to set a horizontal line, representing 5% of the MVC EMG for that respective muscle, on a monitor in front of the subject to serve as visual feedback. The 5% MVC value was utilized in order to reduce the potential fatiguing effect of a sustained contraction. In order to make the visual feedback display easier to follow, the subjects’ active, real time EMG activity level for the tested muscle was rectified and smoothed over a 100ms window. During the experimental testing, subjects were instructed to match the 5% MVC line with their EMG activity for a given target muscle by contracting that specific muscle against the vertical handle. Care was taken to ensure that the subjects produced an isolated muscle contraction with minimal activation through investigators discretion, from the supporting musculature. This was ensured through proper placement of the subject’s arm and careful monitoring of the associated EMG. If the subject was found to have trouble isolating a given muscle contraction and have large amount of synergistic muscle activation, the subject was asked to relax and try again with a better focus on muscle contraction isolation. As the subject maintained this muscle contraction, they were stimulated (Digit II, V, and II+III stimulation) (Fig 1D) as described below, to evoke the CSP response within that muscle.

Each subject was tested under three different stimulation conditions that were performed in random order. The conditions consisted of stimulating the intermediate phalanges of: 1) Digit II (innervated by median and radial nerves) (Fig 1D:1), 2) Digit V (innervated by ulnar nerve) (Fig 1D:3), and 3) Digit II with a conditioning, pre-pulse stimulation of Digit III (innervated by median and radial nerves) (Fig 1D:2) during the low intensity voluntary contractions. Independent stimulation of Digits II and V allowed the investigation of the reflex response to noxious stimulation arising from different nerve pathways. Additionally, the possibility exists that sensory input in conjunction with noxious stimulation may result in different reflex response profiles, as seen in other pre-pulse inhibition studies [15]. Therefore, the additional conditioning stimulation condition was also investigated. The combining of these different stimulation parameters allowed for a more robust investigation of the CSP response within the muscles of the upper limb. All stimulation conditions were tested for each muscle during their individual contractions resulting in data for all muscles and stimulation conditions Each subject performed the three conditions in the same testing session with 90 seconds of rest provided between conditions. At the conclusion of the testing, the distance (cm) from the spinous process C7 to the center of each single differential EMG electrode at its individual site of placement was taken using cloth measuring tape contouring around the upper limb to each electrode (Fig 1C:1–7). This measurement allowed for a general distance indicator for each electrode, representing the muscle location, from the spinous process C7 to generally test potential differences within reflex response as a measure of general distance from the spinal cord.

#### Stimulation parameters

Noxious electrical stimulation (square-wave pulses, 0.5ms duration) was delivered to digits II, V, and II+III using ring electrodes (distal ring–anode; proximal ring–cathode) secured around the intermediate phalanges of the right hand (Fig 1D). Sensory (perceptual) threshold was determined by slowly increasing the electrical current used for stimulation until perceivable by the subject. Perceptual threshold was determined as the lowest stimulation intensity in which the subject could correctly identify 5 out of 10 randomly delivered stimuli. The level of stimulus intensity utilized during the experiment was set at 10x perceptual threshold. This resulted in a stimulus intensity typically between 30–50 mA for most subjects, which is consistent with other studies in the upper limb [2–4, 12, 16, 17]. For the conditioned stimulation, subjects received a stimulation set at perceptual threshold on digit III followed by a noxious stimulation of digit II 100ms later. Subjects were stimulated (0.5Hz ± 0.2Hz stimulation rate) for a total of 20 stimuli within 3 digit stimulation parameters for each of the 7 muscles leading to a total of 420 stimuli (7 muscles x 3 conditions x 20 stimuli).

#### Data analysis

**Fig 2:**
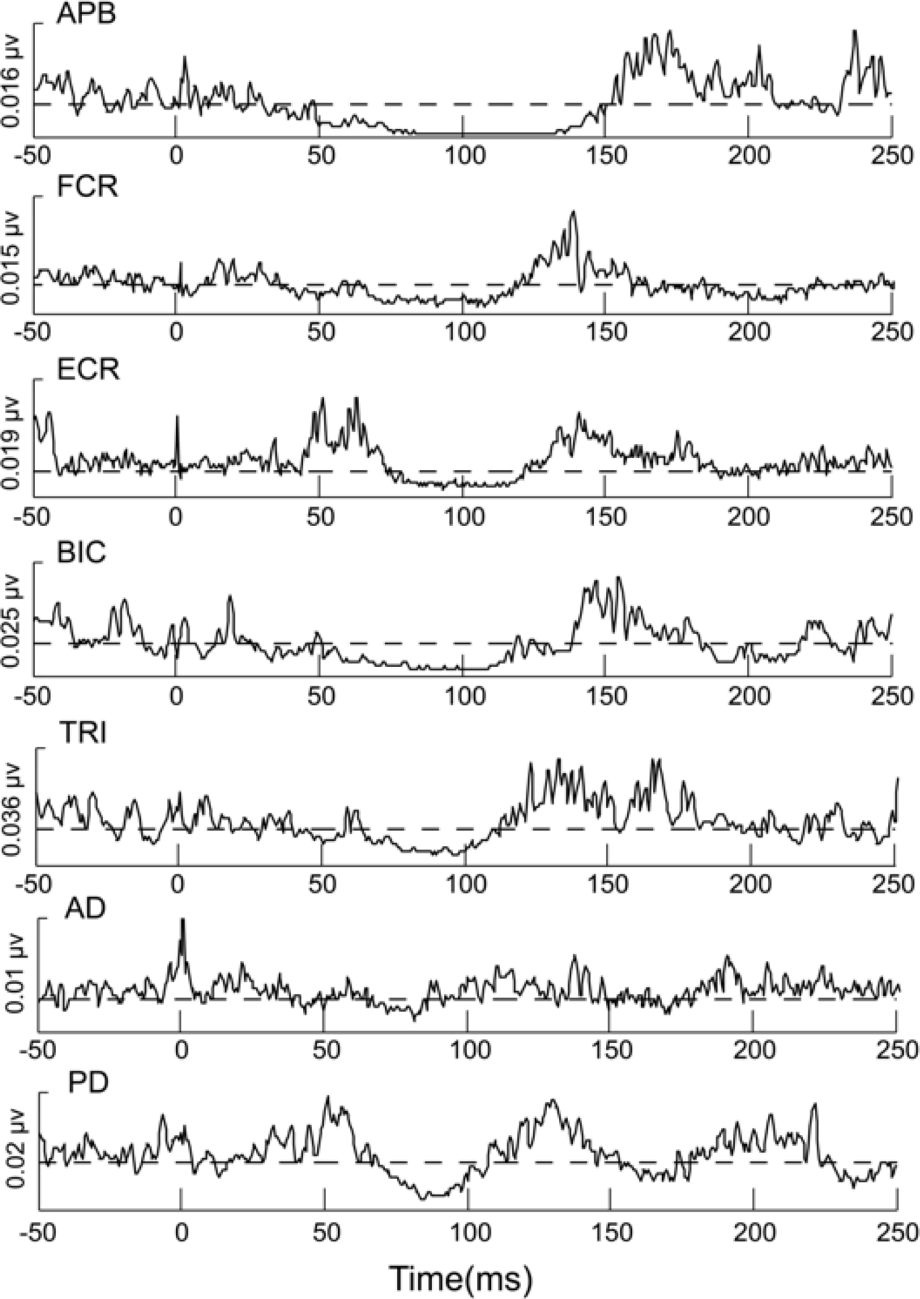
Representative data from one subject averaged across 20 stimulations for all the recorded muscles in the right upper limb. The dashed line represents 80% of the background EMG averaged over 100ms pre-stimulus. The time window is from 50ms prior-to 250ms after digit stimulation.

Surface EMG signals were amplified, conditioned with high- and low-pass cut-off frequencies of 30Hz and 500Hz, recorded at 2,000Hz, and stored at a final gain of 1,000x. Each of the EMG signals were rectified and processed through a custom algorithm written in MATLAB (Mathworks, MA, USA). The custom MATLAB program allowed for the CSP response, for each of the individual stimuli to be identified, individual reflex response phases quantified (i.e. any preceding (E1) or following (E2) excitatory responses to the CSP inhibitory response), and subsequently averaged for analysis. Additionally, the custom algorithm was written within MATLAB to determine the average level of background EMG activity from a 100ms pre-stimulus baseline period (-100ms—stimulation), from which a superimposed horizontal line representing an 80% decrease of this mean EMG activity was placed to determine a suppression of EMG activity from baseline [4]. This process ensured reproducibility across trials for both visual identification and quantification of the individual phases (Fig 1E) within the reflex response (Fig 2). Individual inhibitory and excitatory (E2) phases were identified within each trials reflex response for each stimulation condition and muscle allowing for clear identification of the inhibitory period. This became necessary as previous investigations have reported responses to cutaneous stimulation with periods of fluctuating excitatory (i.e. E1, E2) and inhibitory responses (CSP) [18]. The inhibitory period was identified as the point where EMG activity dropped below the 80% baseline EMG activity (onset) and then returned back to 80% baseline (offset) [4]. This suppressed period of EMG was considered the inhibitory period as long as the duration of the suppressed EMG was greater than 5ms. The time points for the onsets and offsets for each phase of the reflex response were visually identified, by investigators, as the intersection of the superimposed horizontal line of the mean EMG activity recorded with the raw EMG trace. From the onset and offset data, the individual phase durations were calculated by subtracting the onset time from the offset time. In addition, the percentage of inhibition or excitation was calculated by dividing the level of EMG activity during the individual phase (inhibition or E2) by the baseline EMG amplitude calculated from the 100ms pre-stimulus window[4, 5, 12, 15]. The individual trial EMG rebound (E2) was identified as the point in which the mean level of EMG activity returned and subsequently increased above the 80% baseline previously calculated. This mean level rebound activity was quantified over a 100ms period of EMG activity starting at the point of EMG return to baseline [15]. All unquantifiable phases within the CSP response were eliminated from analysis.

#### Statistical analysis

Statistical analyses were performed utilizing the statistics toolbox in MATLAB (Mathworks, MA, USA). Initial analysis identified two violations within the data (normality and sphericity). Analysis of kurtosis determined the deviation from normality to be within acceptable range (i.e. less than -1 or greater than 2), and Greenhouse-Geisser correction was used to correct the sphericity violation. A period of background EMG for each muscle was initially analyzed via one-way ANOVA to determine EMG consistency across data collection. Three-way ANOVAs were used to compare the onsets, offsets, duration of inhibition, suppression level of EMG, and EMG rebound across the three stimulation conditions (Digit II, V, and III+II) (Fig 1D: 1–3) within each muscle. Post-Hoc tests utilizing the Bonferroni model were conducted in order to determine the direction of any individual differences that occurred. Linear regressions were used to determine any significant relationships that may have occurred between within the CSP response with the distance of the muscle from the spinal cord. All results were considered significant at *p* < 0.05.

## Results

**Fig 3:**
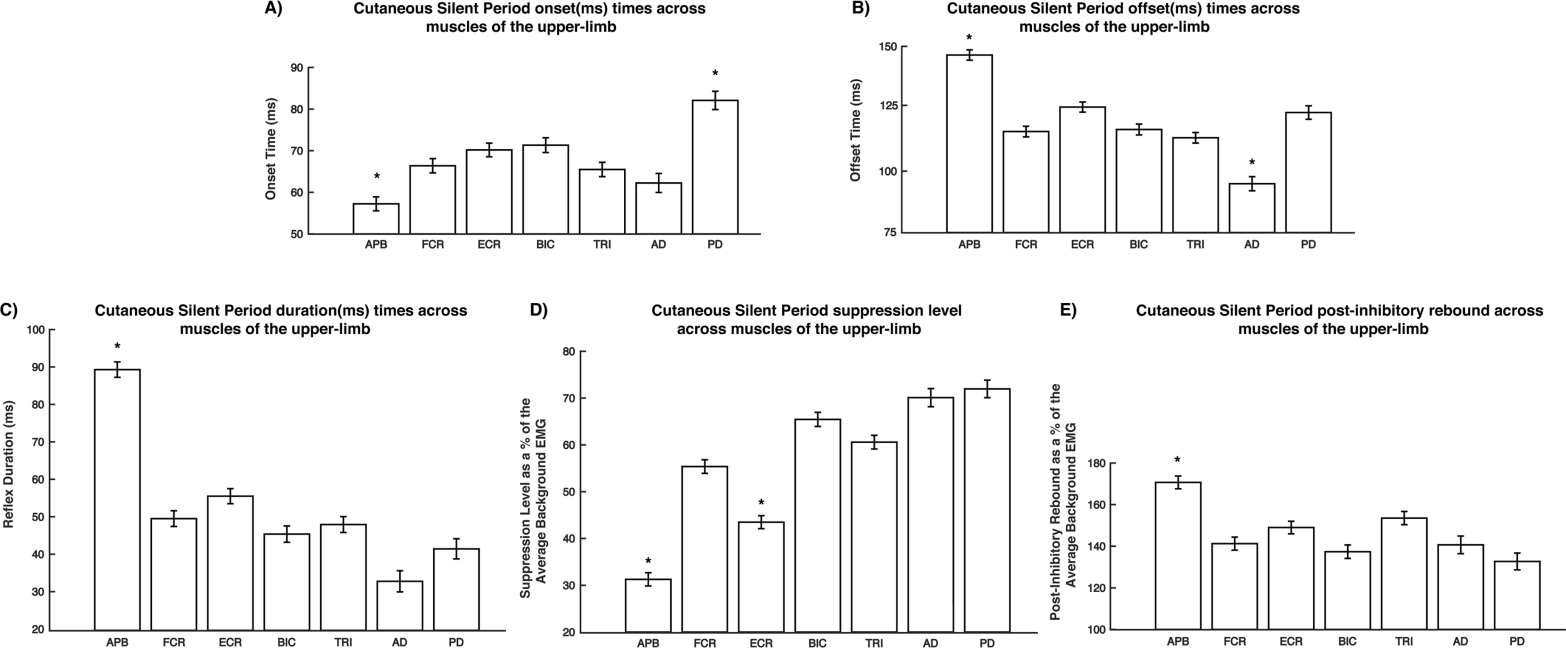
Results (mean ± std) collapsed across stimulation conditions for all muscles for A) reflex onset time, B) reflex offset time, C) reflex duration, D) level of suppression, and E) post-inhibitory rebound. Results were considered significant if p < 0.05. * Denotes significance.

All voluntarily activated muscles demonstrated the CSP response (see Fig 3). Preliminary analysis of the background EMG (100 – 50ms pre-stimulus) resulted in no significant differences across muscles (F_(6,147_) = 0.53, *p* = 0.79). Three-way ANOVA’s for each of the dependent variable across stimulation conditions within each muscle tested, resulted in no significant differences (see Table 1). Therefore, all comparisons were made on the collapsed data for the stimulation conditions to determine potential differences across the muscles tested. Descriptive data on the 22 subjects for onset times, reflex durations, reflex suppression, and post-inhibitory rebound for all conditions can be found in Table 2.

**Table 1:** Statistical results comparing each dependent variable across stimulations within each muscle tested.

	Onset (ms)	Offset (ms)	Duration (ms)	Suppression (% Baseline)	EMG Rebound (% Baseline)
**APB**	F_(2,61)_ = 1.21, *p* = 0.31	F_(2,61)_ = 0.14, *p* = 0.87	F_(2,61)_ = 0.16, *p* = 0.86	F_(2,61)_ = 1.41, *p* = 0.25	F_(2,61)_ = 0.26, *p* = 0.78
**FCR**	F_(2,60_) = 1.35, *p* = 0.27	F_(2,58)_ = 0.22, *p* = 0.80	F_(2,58)_ = 0.51, *p* = 0.60	F_(2,58)_ = 0.46, *p* = 0.64	F_(2,58)_ = 1.07, *p* = 0.35
**ECR**	F_(2,63_) = 0.60, *p* = 0.55	F_(2,63)_ = 1.05, *p* = 0.35	F_(2,63)_ = 1.76, *p* = 0.18	F_(2,63)_ = 0.79, *p* = 0.46	F_(2,63)_ = 2.39, *p* = 0.10
**BIC**	F_(2,54_) = 0.77, *p* = 0.47	F_(2,54)_ = 0.45, *p* = 0.64	F_(2,54)_ = 0.06, *p* = 0.94	F_(2,54)_ = 0.86, *p* = 0.43	F_(2,54)_ = 0.24, *p* = 0.79
**TRI**	F_(2,57_) = 0.07, *p* = 0.93	F_(2,57)_ = 0.55, *p* = 0.58	F_(2,57)_ = 0.07, *p* = 0.94	F_(2,57)_ = 0.05, *p* = 0.96	F_(2,57)_ = 0.85, *p* = 0.43
**AD**	F_(2,31_) = 0.63, *p* = 0.54	F_(2,31)_ = 0.18, *p* = 0.84	F_(2,31)_ = 0.40, *p* = 0.67	F_(2,31)_ = 1.93, *p* = 0.16	F_(2,31)_ = 0.05, *p* = 0.96
**PD**	F_(2,34_) = 0.04, *p* = 0.96	F_(2,34)_ = 0.47, *p* = 0.63	F_(2,34)_ = 0.99, *p* = 0.38	F_(2,34)_ = 0.31, *p* = 0.73	F_(2,34)_ = 0.97, *p* = 0.39

**Table 2:** Average values ± standard deviations for the inhibitory reflex onsets, durations, suppressions, and post-inhibitory rebounds for each stimulation condition across all muscles.

	Onset (ms)	Duration (ms)	Suppression (% Baseline)	EMG Rebound (% Baseline)
**Digit II**	66.7 ± 11.4	51.9 ± 13.3	55.9 ± 10.9	148.9 ± 20.1
**Digit V**	69.9 ± 12.4	50.3 ± 14.2	58.6 ± 11.4	147.6 ± 20.7
**Digit II+III**	67.3 ± 13.6	52.5 ± 16.5	56.5 ± 10.5	142.9 ± 18.4

### Inhibitory reflex analysis

**Fig 4:**
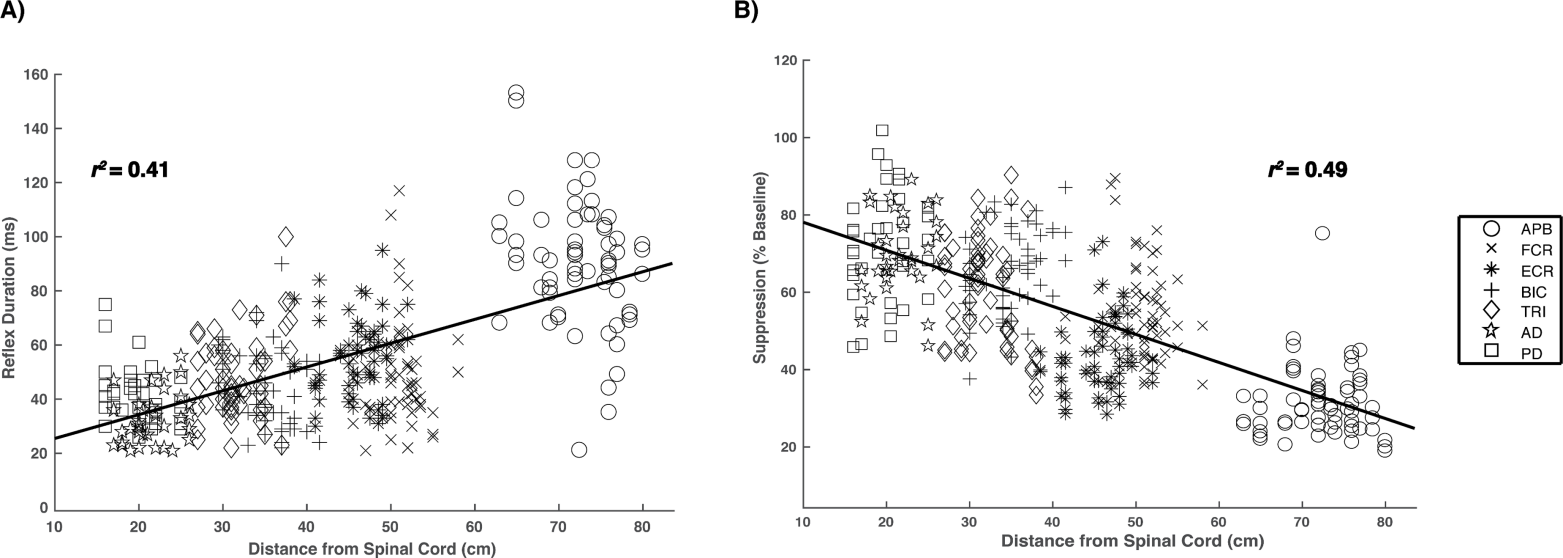
Regression results comparing the inhibitory reflex durations (ms) and level of suppression (% of baseline) with the distance of each muscle from the spinal cord.

Collapsing across stimulations and looking across muscles resulted in significant main effects (*p* < 0.001) for onset time (F_*(6*,*372)*_ = 16.1), offset time (F_*(6*,*372)*_ = 44.47), reflex duration (F_*(6*,*372)*_ = 66.96), reflex suppression (F_*(6*,*372)*_ = 92.53), and post-inhibitory rebound (F_*(6*,*372)*_ = 15.02) (Fig 4). Descriptive data on the 22 subjects for background EMG, onset times, reflex durations, reflex suppression, and post-inhibitory rebound for all muscles can be found in Table 3. On average the APB muscle demonstrated the earliest onset time, latest offset times and therefore longest duration, smallest level of reflex suppression, and largest post-inhibitory rebound compared with the other muscles. In contrast, the proximal muscles of the shoulder demonstrated the latest onset time (Fig 3A), shortest reflex durations (Fig 3C), and one of the largest reflex suppressions (Fig 3D). Comparison of the dependent variables across muscles can be found in Fig 3.

**Table 3:** Average values ± standard deviations for the background EMG, inhibitory reflex onsets, durations, suppressions, and post-inhibitory rebounds across all muscles.

	Background EMG (mV)	Onset (ms)	Offset (ms)	Durations (ms)	Suppression (%baseline)	EMG Rebound (%baseline)
**APB**	0.0041±2.79e-06	57.2±1.7	146.5±2.1	89.3±2.1	31.3±1.4	170.7±3.1
**FCR**	0.0064±5.39e-06	66.4±1.7	115.9±2.1	49.5±2.1	55.4±1.4	141.2±3.2
**ECR**	0.0026±2.15e-06	70.2±1.6	125.7±2.0	55.5±2.0	43.5±1.4	149.0±3.0
**BIC**	0.0031±1.89e-06	71.3±1.8	116.7±2.2	45.4±2.2	65.4±1.5	137.4±3.3
**TRI**	0.0043±2.52e-06	65.5±1.7	113.4±2.1	47.9±2.1	60.6±1.5	153.5±3.2
**AD**	0.0080±5.03e-06	62.2±2.3	95.0±2.8	32.8±2.8	70.1±1.9	140.7±4.2
**PD**	0.0175±0.00e-06	82.1±2.2	123.5±2.7	41.4±2.7	72.0±1.9	132.7±4.0

### Linear regression analysis

Linear regression analysis resulted in a low-to-moderate relationship between the muscle distance from the spinal cord with both CSP duration and suppression level (Fig 4). This result was present across all stimulation conditions. Therefore, the general anatomical distance of a muscle from the spinal cord (cm) may serve as an indicator for CSP duration (ms) and level of suppression (% of baseline EMG). Specifically, the further the distance, the longer and shallower the evoked CSP will be regardless which digit was stimulated (*p* < 0.001).

## Discussion

The current study sought to investigate potential alterations in the CSP nociceptive reflex by addressing 2 aims: 1) primarily investigate the CSP response across multiple extensor and flexor muscles of the upper limb, and 2) secondarily investigate the influence of stimulation site on the reflex response. The current study demonstrated a clear presence of the CSP response within both flexor and extensor muscles of the upper limb, directly addressing aim #1. Additionally, the study suggests that stimulation site (i.e. Digit II, V, and II/III stimulation) does not play a role in influencing the reflex response, addressing aim #2. Such results provide a rational for further investigations on the intricacies of peripheral nociception processing.

### Aim 1: CSP and extensor and flexor muscles

The primary aim of this study sought to investigate the CSP across multiple extensor and flexor muscles of the upper limb. Early investigations provided direct evidence of the CSP serving as a protective reflex and suggested an interplay between inhibitory and excitatory reflex responses such as inhibition within extensor muscles (i.e. CSP response) and excitation of flexor muscles (i.e. withdrawal reflex) [4, 10, 13, 19, 20]. As data on the hand and forearm became available, this classical activation pattern came into question as inhibition of the hand muscles was present for both flexor and extensor muscles [3, 10, 11]. These reports, however, only provided isolated instances of the CSP response within a few individual muscles rather than across the multiple muscles of the upper limb. The present study, however, was able to demonstrate the existence of the inhibitory CSP response within both flexor and extensor muscles of the upper limb, confirming previous isolated hand muscle recordings while also expanding the CSP response across multiple muscles of the upper limb regardless of their specific task (i.e. flexion or extensor). As a result, the current study suggests that the CSP inhibits voluntarily activated muscle regardless of their immediate function. This blanket inhibition may be the “first line of defense” during the navigation and exploration of potentially hazardous environments (i.e. reaching) by halting the immediate action that may introduce harm to the limb.

### Aim 2: Stimulation site

The secondary aim of the current study was to investigate the influence of multiple stimulation sites on the reflex response. Through stimulation of various digits of the hand supplied by different afferents, the current study was able to demonstrate that different stimulation sites played no role in altering the CSP response within the muscles of the upper limb. This result strengthens the previous assertion that the CSP inhibits voluntarily activated muscle, in this case, regardless of the stimulation.

### CSP response alterations

Further investigation into the individual components of the CSP response (i.e. onset, offset, duration, etc.) revealed altered response patterns based on the associated muscles approximate location within the upper limb. Specifically, the distal muscles of the upper limb presented with the earliest reflex onset times, latest offset times, longest reflex duration, and lowest level of EMG suppression when compared to the more proximal muscles of the shoulder. Theoretically, the pronounced CSP within these distal muscles would allow for increased opportunity for movement adjustment within those muscles, when exposed to noxious stimulation. The results of the current study support this contention and are in accord with distal muscle data and kinematic reports presented by previous investigations [4, 11, 21, 22]. The pathways associated with such adjustments are well known as sensory inputs from the hand are known to exert effects on a large proportion of motor cortex neurons related to hand movement [23, 24], by converging on common spinal interneurons with corticospinal tract fibers [25–27]. These results give greater validity to the concept that strong inhibitory control of distal muscles is needed to allow for complex manipulative and exploratory tasks, especially within a potentially hazardous environment where injury may occur [4, 8, 28, 29]. This concept is anatomically supported by distinctly different and direct cortico-motoneuronal projections to the hand, forearm, and upper arm muscles [30–32]. The longer inhibitory periods occurring within the APB of the hand may be a product of these direct cortico-motoneuronal projections allowing for a greater period of time for manipulative corrections in the presence of noxious stimuli. Along these lines the differences occurring within the CSP response across the upper limb muscles may be a product of the differing cortico-motoneuronal projections, as greater time periods for corrective movements in response to noxious stimulation may not be as greatly needed. As it stands, the current study results, when paired with previous investigations, suggest the potential for a distal to proximal relationship for the CSP response. Therefore, it may be suggested that the current understanding of nociceptive input processing needs revision.

## Conclusions

The results presented within the current study suggest that the classical pattern associated with the inhibitory CSP response (i.e. pure inhibition of extensors only) during voluntary muscle contraction within the upper limb requires revision. The main findings of the current study point to a potential differential level of spinal nociceptive processing existing between the distal and proximal muscles of the upper limb. Therefore, the level of spinal processing of nociceptive input appear more complex than once believed and requires further investigation. While the precise mechanisms of the inhibitory CSP response remain unclear, the present study provides a refinement within the current understanding of the spinal organization associated with nociception input processing and the associated motor control of the upper limb.
